# Supplementary material for: Health outcomes in hospitalised and non-hospitalised individuals after COVID-19, an observational, cross-sectional study
Source: Commun Med (Lond). 2025 Dec 4;5:512. doi: 10.1038/s43856-025-01251-5 (PMC12678783; doi:10.1038/s43856-025-01251-5)
Supplement: Supplementary file 2 — Supplementary Materials [file 43856_2025_1251_MOESM2_ESM.pdf]

Supplementary Materials

Supplementary Table S1. Clinical outcomes at the follow-up assessment after COVID-19 of the total cohort participants, also presented as Non-Hospitalised group (NH-group) and Hospitalised group (H-group).

Supplementary Table S2. Clinical outcomes at the follow-up assessment after COVID-19 presented by cluster 1 to 4, and for the total cohort.

Supplementary Table S1. Clinical outcomes at the follow-up assessment after COVID-19 of the total cohort participants, also presented as Non-Hospitalised group (NH-group) and Hospitalised group (H-group).

Clinical outcomes	Total (n=931)	NH-group (n=449)	H-group (n=482)	MD (95% CI)
**Physical function**<br>Objectively Measured				
**6MWT,** distance in meters				
Mean (SD)	478 (140)	482 (136)	474 (145)	7.95 (-10.34; 26.25)<br>0.052
Missing, n (%)	25 (2.7%)	5 (1.1%)	20 (4.1%)	
**1MSTST test,** repetitions				
Mean (SD)	25 (11)	27 (11)	23 (10)	3.32 (1.93; 4.72)<br>1.98e-06
Missing, n (%)	53 (5.7%)	26 (5.8%)	27 (5.6%)	
**Grip strengt**[h], kg				
Mean (SD)	32 (11)	30 (10)	33 (13)	-2.88 (-4.36; -1.41)<br>7.43e-05
Missing, n (%)	54 (5.8%)	33 (7.3%)	21 (4.4%)	
**Lung function**				
**FVC,** in liters				
Mean (SD)	3.52 (0.91)	3.72 (0.81)	3.33 (1.0)	0.39 (0.27; 0.51)<br>3.24e-10
Missing	77 (8.3%)	37 (8.3%)	40 (8.3%)	
**FEV_1,** in seconds				
Mean (SD)	2.78 (0.74)	2.93 (0.67)	2.64 (0.77)	0.29 (0.2; 0.39)<br>1.09e-08
Missing	77 (8.3%)	37 (8.3%)	40 (8.3%)	
**FEV1/FVC ratio**				
Mean (SD)	0.79 (0.08)	0.79 (0.07)	0.80 (0.08)	-0.01 (-0.02; 0.00)<br>0.053
Missing, n (%)	77 (8.3%)	37 (8.2%)	40 (8.3%)	
**MIP**, cmH_2O				
Mean (SD)	83.7 (31.1)	79.3 (27.2)	87.9 (33.9)	-8.66 (-12.64; -4.68)<br>5.51e-05
Missing, n (%)	18 (1.9%)	4 (0.9%)	14 (2.9%)	
**Mental Health, cognitive function and fatigue**<br>Self-assessed				
**PHQ-9 depression symptom**				
Median (IQR)	9.0 (4.0 to 13.0)	11.0 (7.0 to 15.0)	6.0 (2.0 to 12.0)	5 (4; 6)<br>2.65e-19
Missing	88 (9.5%)	22 (4.9%)	66 (13.7%)	
**GAD-7 anxiety symptom**				
Median (IQR)	4.5 (1.0 to 9.0)	5.0 (2.0 to 9.0)	4.0 (1.0 to 9.0)	1 (0; 2)<br>0.001
Missing	85 (9.1%)	19 (4.2%)	66 (13.7%)	
**MoCA**				
Median (IQR)	26.0 (24.0 to 28.0)	27.0 (25.0 to 28.0)	25.0 (22.0 to 27.0)	2 (1; 2)<br>6.80e-16
Missing	224 (24.1%)	130 (29.0%)	94 (19.5%)	
**FSS fatigue' severity**				
Median (IQR)	53.0 (39.0 to 60.0)	57.0 (51.0 to 61.0)	41.0 (24.0 to 53.0)	16 (13; 21)<br>2.65e-31

Missing	365 (39.2%)	115 (25.6%)	250 (51.9%)	

Data are presented as numbers (%), mean (SD), or median (IQR) and mean or median differences (MD) at a 95% confidence interval (CI)[1] using t-test or the Wilcoxon rank-sum test for continuous variables and Chi-squared or Fisher´s exact test for categorical variables depending on data level. A two-sided p-value < 0.05 was considered statistically significant[2]. Abbreviations: 6MWT=Six-Minute Walking Test. 1MSTST=1 minute sit-to-stand test. FVC=forced vital capacity. FEV1=forced expiratory volume in one second. MIP=Maximal Inspiratory Pressure. PHQ-9= the Patient Health Questionnaire-9. GAD-7=General Anxiety Disorder Questionnaire-7. MoCA= Montreal Cognitive Assessment test. FSS=Fatigue Severity Scale

Supplementary Table S2. Clinical outcomes at the follow-up assessment after COVID-19 presented by cluster 1 to 4, and for the total cohort.

	**Cluster 1 n=111**	**Cluster 2 n=164**	**Cluster 3 n=236**	**Cluster 4 n=259**	**Total n=770**
**Physical function** Objectively measured					
**6MWT,** distance in meters					
Mean (SD)	301 (110)	526 (96)	439 (107)	576 (92)	484 (137)
**1MSTST test,** repetitions					
Mean (SD)	15 (6)	28 (8)	21 (7)	32 (10)	25 (10)
**Grip strength** kg					
Mean (SD)	26 (11)	32 (11)	29 (10)	36 (11)	32 (11)
Missing, n (%)	16 (14.4%)	19 (11.6%)	34 (14.4%)	28 (10.8%)	97 (12.6%)
**Lung function**					
**FVC,** in liters					
Mean (SD)	3.36 (0.84)	3.63 (0.97)	3.45 (0.90)	3.72 (0.89)	3.57 (0.91)
Missing	11 (9.9%)	10 (6.1%)	24 (10.2%)	21 (8.1%)	66 (8.6%)
**FEV_1,** in seconds					
Mean (SD)	2.67 (0.71)	2.86 (0.76)	2.75 (0.73)	2.90 (0.71)	2.81 (0.73)
Missing	11 (9.9%)	10 (6.1%)	24 (10.2%)	21 (8.1%)	66 (8.6%)
**FEV1/FVC ratio**					
Mean (SD)	77.9 (16)	84.1 (17)	79.6 (16)	85.5 (15)	82.3 (16)
Missing, n (%)	11 (9.9%)	10 (6.1%)	24 (10.2%)	21 (8.1%)	66 (8.6%)
**MIP** cmH2O					
Mean (SD)	64.7 (25.7)	90.7 (27.3)	73.9 (27.5)	99.3 (28.5)	85 (31)
**Mental Health, cognitive function and fatigue** Self-assessed					
**PHQ-9 depression symptom,** n=833					
Median (IQR)	16.0 (13.0 to 20.0)	14.0 (12.0 to 18.0)	6.0 (3.0 to 9.0)	4.0 (2.0 to 8.0)	9.0 (4.0 to 13.0)
**GAD-7,** n=834					
Median (IQR)	11.0 (8.0 to 15.0)	10.5 (8.0 to 15.0)	3.0 (1.0 to 5.0)	1.0 (0.0 to 4.0)	4.0 (1.0 to 9.0)
**MoCA,** n=710					
Median (IQR)	25.0 (22.0 to 27.0)	26.0 (24.0 to 28.0)	26.0 (23.8 to 27.0)	26.0 (25.0 to 28.0)	26.0 (24.0 to 28.0)
**FSS fatigue' severity,** n=558					
Median (IQR)	60.0 (55.8 to 62.3)	57.0 (48.0 to 62.0)	51.0 (41.0 to 59.0)	42.0 (26.0 to 55.0)	53.0 (38.8 to 60.0)

Data are presented as numbers (%), mean (SD) or median (IQR). Missing data are only presented for those without full dataset. Abbreviations: 6MWT=Six-Minute Walking Test. 1MSTST=1 minute sit-to-stand test. FVC=forced vital capacity. FEV1=forced expiratory volume in one second. MIP=Maximal Inspiratory Pressure. PHQ-9= the Patient Health Questionnaire-9. GAD-7=General Anxiety Disorder Questionnaire-7. MoCA= Montreal Cognitive Assessment test. FSS=Fatigue Severity Scale
