# Supplementary material for: Health outcomes in hospitalised and non-hospitalised individuals after COVID-19, an observational, cross-sectional study
Source: Commun Med (Lond). 2025 Dec 4;5:512. doi: 10.1038/s43856-025-01251-5 (PMC12678783; doi:10.1038/s43856-025-01251-5)
Supplement: Supplementary file 3 — Description for all supplementary data files [file 43856_2025_1251_MOESM3_ESM.pdf]

Description of additional supplementary file

File name: Supplementary Data 1

Description: Characteristics presented as Demographics, Health-related variables pre COVID-19, and Hospital stay during COVID-19 of the total cohort participants, also presented as Non-Hospitalised (NH-group) and Hospitalised (H-group) groups.

File name: Supplementary Data 2

Description: All symptoms reported at follow up assessment, after COVID-19 and presented as prevalence by Total cohort (n=931), NH-group (n=449) and H-group (n=482).

File name: Supplementary Data 3

Description: Clinical and functional outcomes, symptom burden and self-assessed health pre and post COVID-19 at follow-up in the total cohort and also presented as Non-Hospitalised group (NH-group) and Hospitalised group (H-group).

File name: Supplementary Data 4

Description: Characteristics presented as Demographics, Health-related variables pre COVID-19 and Hospital stay during COVID-19. Clinical and functional outcomes, symptom burden and self-assessed health pre and post COVID-19 at follow-up presented by cluster 1 to 4 and for the total cohort.

File name: Supplementary Data 5

Description: All symptoms reported at follow up assessment, after COVID-19 and presented as prevalence by cluster 1 to 4 (n=770).
