# Supplementary material for: Health outcomes in hospitalised and non-hospitalised individuals after COVID-19, an observational, cross-sectional study
Source: Commun Med (Lond). 2025 Dec 4;5:512. doi: 10.1038/s43856-025-01251-5 (PMC12678783; doi:10.1038/s43856-025-01251-5)
Supplement: Supplementary file 4 — Supplementary Data 1 [file 43856_2025_1251_MOESM4_ESM.docx]

**Supplementary Data 1**

**Characteristics presented as Demographics, Health-related variables pre COVID-19, and Hospital stay during COVID-19 of the total cohort participants, also presented as Non-Hospitalised (NH-group) and Hospitalised (H-group) groups.**

|  | **Total (n=931)** | **NH-group (n=449)** | **H-group (n=482)** |
| --- | --- | --- | --- |
| **Demographics** | | | |
| **Sex** |  |  |  |
| Female | 516 (55.4%) | 379 (84.4%) | 137 (28.4%) |
| Male | 415 (44.6%) | 70 (15.6%) | 345 (71.6%) |
| **Age, years** |  |  |  |
| Mean (SD) | 50.7 (13.1) | 44.4 (11.1) | 56.7 (12.1) |
| **Education** |  |  |  |
| Compulsory School | 39 (4.2%) | 2 (0.4%) | 37 (7.7%) |
| Upper Secondary School | 82 (8.8%) | 27 (6.0%) | 55 (11.4%) |
| Higher education | 516 (55.4%) | 338 (75.3%) | 178 (36.9%) |
| Missing | 294 (31.6%) | 83 (18.5%) | 211 (43.9%) |
| **Occupational status,** pre COVID-19 |  |  |  |
| Retired | 122 (13.1%) | 13 (2.9%) | 109 (22.6%) |
| Sick leave | 32 (3.4%) | 16 (3.6%) | 16 (3.3%) |
| Student | 36 (3.9%) | 28 (6.2%) | 8 (1.7%) |
| Work (fulltime or parttime job) | 707 (75.9%) | 388 (86.4%) | 319 (66.2%) |
| Missing | 34 (3.7%) | 4 (0.9%) | 30 (6.2%) |
| **Health-related variables pre COVID-19** | | | |
| **BMI,** kg/m2 |  |  |  |
| Mean (SD) | 27.9 (5.9) | 25.5 (5.0) | 30.3 (5.9) |
| Missing | 7 (0.8%) | 0 (0.0%) | 7 (1.5%) |
| **Tobacco use** |  |  |  |
| Current smoker | 25 (2.7%) | 13 (2.9%) | 12 (2.5%) |
| Former smoker | 326 (35.0%) | 110 (24.5%) | 216 (44.8%) |
| Never smoked | 541 (58.2%) | 323 (71.9%) | 218 (45.2%) |
| Missing | 39 (4.2%) | 3 (0.7%) | 36 (7.5%) |
| **Comorbidity** |  |  |  |
| 0 | 268 (28.8%) | 163 (36.3%) | 105 (21.8%) |
| 1 | 280 (30.0%) | 155 (34.5%) | 125 (25.9%) |
| ≥2 | 383 (41.1%) | 131 (29.2%) | 252 (52.3%) |
| Hypertension | 248 (26.6%) | 52 (11.4%) | 196 (41.5%) |
| Respiratory diseases | 175 (18.8%) | 75 (16.7%) | 100 (20.7%) |
| Psychiatric conditions | 155 (16.7%) | 116 (25.8%) | 39 (8.1%) |
| Cardiovascular conditions | 77 (8.3%) | 16 (3.6%) | 61 (12.7%) |
| Thyroid diseases | 75 (8.1%) | 42 (9.4%) | 33 (6.8%) |
| Rheumatic diseases | 44 (4.7%) | 14 (3.1%) | 30 (6.2%) |
| Metabolic diseases | 34 (3.7%) | 5 (1.1%) | 29 (6.0%) |
| Kidney diseases | 27 (2.9%) | 3 (0.7%) | 24 (5.0%) |
| Missing | 18 (1.9%) | 16 (3.6%) | 2 (0.4%) |
| **Hospital stay during COVID-19** | | | |
| **Length of hospital stay, days** |  |  |  |
| Mean (SD) |  |  | 25.3 (19.5) |
| Missing |  |  | 15 (3.1%) |
| **ICU admission** |  |  | 304 (63.1%) |
| Missing |  |  | 19 (3.9%) |
| **Length of ICU stay, days** |  |  |  |
| Mean (SD) |  |  | 12.4 (53) |
| Missing |  |  | 19 (3.9%) |
| **ECMO** |  |  | 12 (2.5%) |
| Missing |  |  | 9 (1.9%) |
| **Invasive ventilation** |  |  | 211 (43.8%) |
| Missing |  |  | 16 (3.3%) |
| **Non-invasive ventilation** |  |  | 185 (38.4%) |
| Missing |  |  | 25 (5.2%) |
| **High-flow oxygen therapy** |  |  | 292 (60.6%) |
| Missing |  |  | 25 (5.2%) |

Data are presented as numbers (%) or mean (SD).

Abbreviations: BMI=Body Mass Index. ICU=Intensive Care Unit. ECMO=Extracorporeal Membrane Oxygenation.
