# Supplementary material for: Health outcomes in hospitalised and non-hospitalised individuals after COVID-19, an observational, cross-sectional study
Source: Commun Med (Lond). 2025 Dec 4;5:512. doi: 10.1038/s43856-025-01251-5 (PMC12678783; doi:10.1038/s43856-025-01251-5)
Supplement: Supplementary file 5 — Supplementary Data 2 [file 43856_2025_1251_MOESM5_ESM.docx]

**Supplementary Data 2**

**All symptoms reported at follow up assessment, after COVID-19 and presented as prevalence by Total cohort (n=931), NH-group (n=449) and H-group (n=482).**

|  | **Total, n=931** | **NH-group, n=449** | **H-group, n=482** |
| --- | --- | --- | --- |
| **Symptoms,** prevalence at assessment n (%) |  |  |  |
| Dyspnoea | 570 (61.2%) | 316 (70.4%) | 254 (52.7%) |
| Fatigue | 592 (63.6%) | 418 (93.1%) | 174 (36.1%) |
| Joint pain | 421 (45.5%) | 238 (53.4%) | 183 (38.1%) |
| Cough | 341 (36.6%) | 182 (40.5%) | 159 (33.0%) |
| Paraesthesia | 347 (37.3%) | 211 (47.0%) | 136 (28.2%) |
| Chest pressure | 347 (37.3%) | 264 (58.8%) | 83 (17.2%) |
| Palpitations | 312 (33.5%) | 267 (59.5%) | 45 (9.3%) |
| Reduced fitness | 308 (33.1%) | 131 (29.2%) | 177 (36.7%) |
| Weight gain | 229 (24.6%) | 160 (35.6%) | 69 (14.3%) |
| Concentration | 291 (31.3%) | 213 (47.4%) | 78 (16.2%) |
| Headache | 261 (28.0%) | 212 (47.2%) | 49 (10.2%) |
| Dizziness | 253 (27.2%) | 206 (45.9%) | 47 (9.8%) |
| Memory issues | 296 (31.8%) | 204 (45.4%) | 92 (19.1%) |
| Intermittent fever | 185 (19.9%) | 160 (35.6%) | 25 (5.2%) |
| Muscular weakness | 195 (20.9%) | 84 (18.7%) | 111 (23.0%) |
| Insomnia | 256 (27.5%) | 166 (37.0%) | 90 (18.7%) |
| Weight loss | 206 (22.1%) | 63 (14.0%) | 143 (29.7%) |
| PEM (post exertional malaise) | 153 (16.4%) | 137 (30.5%) | 16 (3.3%) |
| Dysfunctional breathing | 144 (15.5%) | 106 (23.6%) | 38 (7.9%) |
| Chest pain | 117 (12.6%) | 98 (21.8%) | 19 (3.9%) |
| Brain fog | 149 (16.0%) | 136 (30.3%) | 13 (2.7%) |
| Other symptoms (not listed) | 105 (11.3%) | 61 (13.6%) | 44 (9.1%) |
| Nausea | 102 (11.0%) | 95 (21.2%) | 7 (1.5%) |
| Impaired vision | 107 (11.5%) | 84 (18.7%) | 23 (4.8%) |
| Ageusia | 125 (13.4%) | 89 (19.8%) | 36 (7.5%) |
| Anosmia | 126 (13.5%) | 86 (19.2%) | 40 (8.3%) |
| Blemishes | 72 (7.7%) | 51 (11.4%) | 21 (4.4%) |
| Urticaria | 95 (10.2%) | 66 (14.7%) | 29 (6.0%) |
| Walking difficulties | 61 (6.6%) | 17 (3.8%) | 44 (9.1%) |
| Appetite | 109 (11.7%) | 83 (18.5%) | 26 (5.4%) |
| Worried | 94 (10.1%) | 46 (10.2%) | 48 (10.0%) |
| Diarrhea | 73 (7.8%) | 66 (14.7%) | 7 (1.5%) |
| Tinnitus | 70 (7.5%) | 58 (12.9%) | 12 (2.5%) |
| Sensory sensitivity | 88 (9.5%) | 79 (17.6%) | 9 (1.9%) |
| Dysphagia | 63 (6.8%) | 49 (10.9%) | 14 (2.9%) |
| Voice impairments | 74 (7.9%) | 38 (8.5%) | 36 (7.5%) |
| Depressed | 71 (7.6%) | 36 (8.0%) | 35 (7.3%) |
| Reduced balance | 77 (8.3%) | 30 (6.7%) | 47 (9.8%) |
| Fainting | 46 (4.9%) | 41 (9.1%) | 5 (1.0%) |
| Tremor | 57 (6.1%) | 44 (9.8%) | 13 (2.7%) |
| Pain during deep breathing | 51 (5.5%) | 40 (8.9%) | 11 (2.3%) |
| Nightmares | 53 (5.7%) | 24 (5.3%) | 29 (6.0%) |
| Hair loss | 51 (5.5%) | 21 (4.7%) | 30 (6.2%) |
| Changed breathing pattern | 53 (5.7%) | 47 (10.5%) | 6 (1.2%) |
| Obstipation | 38 (4.1%) | 31 (6.9%) | 7 (1.5%) |
| Malaise | 38 (4.1%) | 38 (8.5%) | 0 (0.0%) |
| Anxiety | 57 (6.1%) | 29 (6.5%) | 28 (5.8%) |
| Urinary tract problems | 45 (4.8%) | 21 (4.7%) | 24 (5.0%) |
| Mood changes | 46 (4.9%) | 14 (3.1%) | 32 (6.6%) |
| Rigor | 36 (3.9%) | 24 (5.3%) | 12 (2.5%) |
| Abdominal pain | 45 (4.8%) | 38 (8.5%) | 7 (1.5%) |
| Swollen joints | 34 (3.7%) | 14 (3.1%) | 20 (4.1%) |
| Slow movements | 20 (2.1%) | 9 (2.0%) | 11 (2.3%) |
| Irregular menstruation | 22 (2.4%) | 20 (4.5%) | 2 (0.4%) |
| Hearing impairments | 18 (1.9%) | 9 (2.0%) | 9 (1.9%) |
| Susceptible to infections | 12 (1.3%) | 7 (1.6%) | 5 (1.0%) |
| Erectile dysfunction | 2 (0.2%) | 1 (0.2%) | 1 (0.2%) |
| Yawning | 3 (0.3%) | 3 (0.7%) | 0 (0.0%) |
| Seizures | 1 (0.1%) | 1 (0.2%) | 0 (0.0%) |
