# Supplementary material for: Health outcomes in hospitalised and non-hospitalised individuals after COVID-19, an observational, cross-sectional study
Source: Commun Med (Lond). 2025 Dec 4;5:512. doi: 10.1038/s43856-025-01251-5 (PMC12678783; doi:10.1038/s43856-025-01251-5)
Supplement: Supplementary file 6 — Supplementary Data 3 [file 43856_2025_1251_MOESM6_ESM.docx]

**Supplementary Data 3**

**Clinical and functional outcomes, symptom burden and self-assessed health pre and post COVID-19 at follow-up in the total cohort and also presented as Non-Hospitalised group (NH-group) and Hospitalised group (H-group).**

|  | **Total (n=931)** | **NH-group (n=449)** | **H-group (n=482)** | **MD 95% CI^1^**  ***p*-value^2^** |
| --- | --- | --- | --- | --- |
| **Follow-up,** assessment (days) |  |  |  |  |
| Mean (SD) | 318 (222) | 460 (216) | 181 (118) | 278.6 (255.8; 301.3)  1.21e-96 |
| Missing | 21 (2.3%) | 1 (0.2%) | 20 (4.3%) |  |
| **COVID-19 related variables** | | | | |
| **Sickleave,** at assessment |  |  |  |  |
| 0% | 247 (26.5%) | 121 (26.9%) | 126 (26.1%) |  |
| 25% | 40 (4.3%) | 37 (8.2%) | 3 (0.6%) |  |
| 50% | 99 (10.6%) | 79 (17.6%) | 20 (4.1%) |  |
| 75% | 58 (6.2%) | 50 (11.1%) | 8 (1.7%) |  |
| 100% | 246 (26.4%) | 138 (30.7%) | 108 (22.4%) |  |
| Missing | 241 (25.8%) | 24 (5.3%) | 217 (44.8%) |  |
| **POTS**, diagnosed post COVID 19 | 148 (15.9%) | 136 (30.3%) | 12 (2.5%) | 1.29e-30 |
| **Symptoms,** at follow-up assessment | | | | |
| Mean (SD) | 8.8 (5.4) | 12.2 (4.7) | 5.67 (3.9) | 4.67e-72 |
| ≤ 2 symptoms, n | 113 (12.1%) | 2 (0.4%) | 111 (23.0%) |  |
| ≥10 symptoms, n | 385 (46.5%) | 318 (70.8%) | 67 (13.9%) |  |
| **Outcome measures at assessment** | | | | |
| **PCFS**, pre COVID-19/before illness onset |  |  |  |  |
| Median (IQR) | 0 (0 – 0) | 0 (0 – 0) | 0 (0 – 0) | 0 (0; 0)  0.20 |
| Missing | 75 (8.1%) | 22 (4.9%) | 53 (11.0%) |  |
| **PCFS**, at assessment |  |  |  |  |
| Median (IQR) | 2 (1 – 3) | 3 (2 – 3) | 1 (0 – 2) | 2 (2; 2)  4.14e-46 |
| Missing | 47 (5.0%) | 16 (3.6%) | 31 (6.4%) |  |
| **EQ Visual Analogue Scale** |  |  |  |  |
| Mean (SD) | 51.8 (22.8) | 40.5 (18.6) | 63.9 (20.6) | -23.4 (-26.1; -20.7)  2.84e-56 |
| Missing | 106 (11.4%) | 24 (5.3%) | 82 (17.0%) |  |
| **mMRC** dyspnoea score | | | | |
| Median (IQR) | 2 [1 – 3) | 2 (1 – 3) | 1 (1 – 2) | 1 (1; 1)  1.36e-09 |
| Missing | 66 (7.1%) | 23 (5.1%) | 43 (8.9%) |  |
| **Physical function**  Self-assessed | | | | |
| **Frändin Grimby,** pre COVID-19/before illness onset |  |  |  |  |
| Median (IQR) | 4 (4 – 5) | 5 (4 - 6) | 4 (3 – 5) | 1 (1; 1)  1.43e-25 |
| Missing | 30 (3.2%) | 4 (0.9%) | 26 (5.4%) |  |
| **Frändin Grimby,** at assessment |  |  |  |  |
| Median (IQR) | 3 (2 – 3) | 2 (2 – 3) | 3 (2 – 4) | -1 (-1; 0)  1.55e-15 |
| Missing | 26 (2.8%) | 7 (1.6%) | 19 (3.9%) |  |
| **Physical function**  Objectively Measured | | | | |
| **SpO_2_ %,** at rest |  |  |  |  |
| Mean (SD) | 98.7 (1.49) | 99.5 (0.95) | 98.0 (1.54) | 1.49 (1.33; 1.66)  1.88e-59 |
| Missing, n (%) | 13 (1.4%) | 2 (0.4%) | 11 (2.3%) |  |
| **Heartrate, beats per minute,** at rest |  |  |  |  |
| Mean (SD) | 79.1 (13.7) | 80.0 (13.3) | 78.3 (14.0) | 1.7 (0.1; 3.5)  0.031 |
| Missing, n (%) | 14 (1.5%) | 4 (1.3%) | 10 (2.1%) |  |
| **6MWT** % of predicted |  |  |  |  |
| Mean (SD) | 82% (24) | 79% (23) | 85% (25) | -6 (-9; -3)  0.0003 |
| Missing, n (%) | 30 (3.2%) | 6 (0.9%) | 24 (5.0%) |  |
| **1 MSTST test** % of predicted |  |  |  |  |
| Mean (SD) | 63% (26) | 65% (26) | 61% (26) | 4 (1; 8)  0.016 |
| Missing, n (%) | 53 (5.7%) | 26 (5.8%) | 27 (5.6%) |  |
| **Gripstrength** % of predicted |  |  |  |  |
| Mean (SD) | 85% (25) | 89% (22) | 83% (26) | 6 (2; 9)  0.001 |
| Missing, n (%) | 142 (15.3%) | 108 (24.1%) | 34 (7.1%) |  |
| **Pulmonary function** | | | | |
| **FVC** % of predicted |  |  |  |  |
| Mean (SD) | 80.4 (16.3) | 87.8 (13.3) | 73.5 (15.8) | 14.3 (12.4; 16.3)  1.15e-41 |
| Missing, n (%) | 93 (9.9%) | 41 (9.1%) | 52 (10.8%) |  |
| **FEV_1_** % of predicted |  |  |  |  |
| Mean (SD) | 81.4 (16.2) | 87.4 (13.8) | 75.7 (16.4) | 11.7 (9.7; 13.8)  1.67e-27 |
| Missing, n (%) | 93 (9.9%) | 41 (9.1%) | 52 (10.8%) |  |
| **FEV1/FVC** ratio of predicted |  |  |  |  |
| Mean (SD) | 0.79 (0.08) | 0.79 (0.07) | 0.80 (0.08) | -4.7 (-6.1; -3.3)  1.43e-10 |
| Missing, n (%) | 98 (10.5%) | 45 (10.0%) | 53 (11.0%) |  |
| **MIP** % of predicted |  |  |  |  |
| Mean (SD) | 89% (30) | 86% (27) | 92% (32) | -6 (-10; -2)  0.002 |
| Missing, n (%) | 18 (1.9%) | 4 (0.9%) | 14 (2.9%) |  |
| **Mental Health, cognitive function and fatigue**  Self-assessed | | | | |
| **PHQ-9 depression symptom**  ≥ 10, n (%) | 377 (40.5%) | 242 (53.9%) | 135 (28.0%) | 1.27e-11 |
| Missing, n (%) | 88 (9.5%) | 22 (4.9%) | 66 (13.7%) |  |
| **GAD-7 anxiety symptom**  ≥ 10, n (%) | 197 (21.2%) | 102 (22.7%) | 95 (19.7%) | 0.82 |
| Missing, n (%) | 85 (9.1%) | 19 (4.2%) | 66 (13.7%) |  |
| **MoCA <**26, n (%) | 414 (44.5%) | 148 (33%) | 266 (55.2%) | 6.33e-11 |
| Missing, n (%) | 224 (24.1%) | 130 (29%) | 94 (19.5%) |  |
| **FSS fatigue severity** ≥4, n (%) | 450 (48.3%) | 313 (69.7%) | 137 (28.4%) | 2.76e-23 |
| Missing, n (%) | 365 (39.2%) | 115 (25.6%) | 250 (51.9%) |  |

Data are presented as numbers (%), mean (SD), or median (IQR) and mean or median differences (MD) at a 95% confidence interval (CI)^1^ using t-test or the Wilcoxon rank-sum test for continuous variables and Chi-squared or Fisher´s exact test for categorical variables depending on data level^2^. A two-sided p-value < 0.05 was considered statistically significant. Follow-up = number of days from illness onset to first assessment. PHQ-9 ≥ 10 indicates clinical depression. GAD-7 ≥ 10 indicate anxiety. MoCA <26 indicate cognitive impairment. FSS ≥4 indicate fatigue. Abbreviations: POTS=postural orthostatic tachycardia syndrome. PCFS= the Post-COVID-19 Functional Status. mMRC= the modified Medical Research Council dyspnoea scale. SpO_2_=peripheral oxygen saturation. 6MWT=6 Minute Walking Test. 1MSTST=1 minute sit-to-stand test. FVC=forced vital capacity. FEV1=forced expiratory volume in one second. MIP=Maximal Inspiratory Pressure. PHQ-9= Patient Health Questionnaire. GAD-7=General Anxiety Disorder Questionnaire. MoCA= Montreal Cognitive Assessment test. FSS=Fatigue Severity Scale.
