# Supplementary material for: Health outcomes in hospitalised and non-hospitalised individuals after COVID-19, an observational, cross-sectional study
Source: Commun Med (Lond). 2025 Dec 4;5:512. doi: 10.1038/s43856-025-01251-5 (PMC12678783; doi:10.1038/s43856-025-01251-5)
Supplement: Supplementary file 7 — Supplementary Data 4 [file 43856_2025_1251_MOESM7_ESM.docx]

**Supplementary Data 4**

**Characteristics presented as Demographics, Health-related variables pre COVID-19 and Hospital stay during COVID-19. Clinical and functional outcomes, symptom burden and self-assessed health pre and post COVID-19 at follow-up presented by cluster 1 to 4 and for the total cohort.**

| **Total n=770** | **Cluster 1**  **n=111 (14.5%)** | **Cluster 2**  **n=164 (21.3%)** | **Cluster 3**  **n=236 (30.6%)** | **Cluster 4**  **n=259 (33.6%)** | **Total**  **N=770** |
| --- | --- | --- | --- | --- | --- |
| **Non-Hospitalised (n=400, 52%)** | 68 (61.3%) | 99 (60.4%) | 122 (52.0%) | 111 (42.9%) | 400 (51.9%) |
| **Hospitalised (n=370, 48%)** | 43 (38.7%) | 65 (39.6%) | 114 (48.0%) | 148 (57.1%) | 370 (48.1%) |
| **Demographics** | | | | | |
| **Sex** |  |  |  |  |  |
| Female | 77 (69.4%) | 98 (59.8%) | 139 (58.9%) | 125 (48.3%) | 439 (57.0%) |
| Male | 34 (30.6%) | 66 (40.2%) | 97 (41.1%) | 134 (51.7%) | 331 (43.0%) |
| **Age, years** |  |  |  |  |  |
| Mean (SD) | 45.5 (12.2) | 49.3 (11.8) | 48.8 (13.0) | 54.1 (12.4) | 50.2 (12.8) |
| **Education** |  |  |  |  |  |
| Compulsory School | 5 (4.5%) | 5 (3.0%) | 13 (5.5%) | 7 (2.7%) | 30 (3.9%) |
| Upper Secondary School | 13 (11.7%) | 15 (9.1%) | 22 (9.3%) | 22 (8.2%) | 72 (9.4%) |
| Higher education | 69 (62.2%) | 92 (56.1%) | 133 (56.4%) | 144 (55.6%) | 438 (57.0%) |
| Missing | 24 (21.6%) | 52 (31.7%) | 68 (28.8%) | 87 (33.6%) | 231 (30.0%) |
| **Occupational status**, pre COVID-19 |  |  |  |  |  |
| Retired | 5 (4.5%) | 11 (6.7%) | 31 (13.1%) | 46 (17.8%) | 93 (12.1%) |
| Sickleave | 6 (5.4%) | 8 (4.9%) | 9 (3.8%) | 4 (1.5%) | 27 (3.5%) |
| Student | 10 (9.0%) | 5 (3.0%) | 10 (4.2%) | 4 (1.5%) | 29 (3.8%) |
| Work (fulltime or parttime job) | 85 (76.6%) | 135 (82.3%) | 181 (76.7%) | 200 (77.2%) | 601 (78.1%) |
| Missing | 5 (4.5%) | 5 (3.0%) | 5 (2.1%) | 5 (1.9%) | 20 (2.6%) |
| **Health-related variables pre COVID-19** | | | | | |
| **BMI kg/m2** |  |  |  |  |  |
| Mean (SD) | 27.7 (6.4) | 28.0 (5.2) | 27.8 (5.7) | 27.8 (6.3) | 27.8 (5.9) |
| **Tobacco use** |  |  |  |  |  |
| Current smoker | 2 (1.8%) | 2 (1.2%) | 10 (4.2%) | 6 (2.3%) | 20 (2.6%) |
| Former smoker | 34 (30.6%) | 65 (39.6%) | 76 (32.2%) | 88 (34.0%) | 263 (34.2%) |
| Never smoked | 72 (64.9%) | 95 (57.9%) | 141 (59.7%) | 155 (59.8%) | 463 (60.1%) |
| Missing | 3 (2.7%) | 2 (1.2%) | 9 (3.8%) | 10 (3.9%) | 24 (3.1%) |
| **Comorbidity** |  |  |  |  |  |
| 0 | 33 (29.7%) | 49 (29.9%) | 74 (31.4%) | 79 (30.5%) | 235 (30.5%) |
| 1 | 30 (27.0%) | 49 (29.9%) | 75 (31.8%) | 74 (28.6%) | 228 (29.6%) |
| ≥2 | 48 (43.2%) | 66 (40.2%) | 87 (36.9%) | 106 (40.9%) | 307 (39.9%) |
| Hypertension | 27 (24.3%) | 43 (26.2%) | 53 (22.5%) | 77 (30.1%) | 200 (26.0%) |
| Respiratory diseases | 26 (23.4%) | 28 (17.1%) | 40 (16.9%) | 50 (19.3%) | 144 (18.7%) |
| Psychiatric conditions | 18 (16.2%) | 46 (28.0%) | 34 (14.4%) | 32 (12.4%) | 130 (16.9%) |
| Cardiovascular conditions | 5 (4.5%) | 11 (6.7%) | 19 (8.0%) | 18 (6.9%) | 53 (6.9%) |
| Thyroid diseases | 16 (14.4%) | 15 (9.2%) | 18 (7.6%) | 16 (6.2%) | 65 (8.4%) |
| Rheumatic diseases | 3 (2.7%) | 11 (6.8%) | 13 (5.5%) | 8 (3.1%) | 35 (4.5%) |
| Metabolic diseases | 5 (4.5%) | 5 (3.1%) | 5 (2.1%) | 10 (3.9%) | 25 (3.2%) |
| Kidney diseases | 2 (1.8%) | 2 (1.2%) | 11 (4.6%) | 4 (1.6%) | 19 (2.5%) |
| Missing | 2 (1.8%) | 4 (2.4%) | 5 (2.1%) | 5 (1.9%) | 16 (2.1%) |
| **Hospital stay during COVID-19** | | | | | |
| **Participants with hospital stay** | (n=43) | (n=65) | (n=114) | (n=148) | n=370 |
| **Length of hospital stay, days** |  |  |  |  |  |
| Mean (SD) | 34.2 (24.1) | 25.7 (15.2) | 30.6 (24.2) | 18.7 (14.3) | 25.2 (19.9) |
| Missing | 3 (7.0%) | 2 (3.1%) | 5 (4.4%) | 0 (0%) | 10 (2.7%) |
| **ICU admission** | 29 (67.4%) | 46 (70.8%) | 77 (67.5%) | 78 (52.7%) | 230 (62.2%) |
| Missing | 5 (11.6%) | 2 (3.1%) | 3 (2.6%) | 1 (0.7%) | 11 (3.0%) |
| **Length of ICU stay**, days  Mean (SD) | 18.3 (17.0) | 11.0 (11.1) | 13.4 (16.1) | 6.14 (9.48) | 10.5 (13.6) |
| **ECMO** | 5 (11.6%) | 1 (1.5%) | 5 (4.4%) | 1 (0.7%) | 12 (3.2%) |
| Missing | 2 (4.7%) | 2 (3.1%) | 0 (0%) | 1 (0.7%) | 5 (1.4%) |
| **Invasive ventilation** | 25 (58.1%) | 35 (53.8%) | 59 (51.8%) | 49 (33.1%) | 168 (45.4%) |
| Missing | 4 (9.3%) | 2 (3.1%) | 3 (2.6%) | 1 (0.7%) | 10 (2.7%) |
| **Non-invasive ventilation** | 14 (32.6%) | 32 (49.2%) | 34 (29.8%) | 48 (32.4%) | 128 (34.6%) |
| Missing | 5 (11.6%) | 3 (4.6%) | 4 (3.5%) | 5 (3.4%) | 17 (4.6%) |
| **High-flow oxygen therapy** | 26 (60.5%) | 37 (56.9%) | 73 (64.0%) | 79 (53.4%) | 215 (58.1%) |
| Missing | 5 (11.6%) | 4 (6.2%) | 4 (3.5%) | 4 (2.7%) | 17 (4.6%) |
| **Clinical and functional outcomes, n=770** | | | | | |
| **Follow-up,** assessment (days) |  |  |  |  |  |
| Mean (SD) | 306 (190) | 341 (228) | 305 (211) | 309 (213) | 309 (213) |
| **COVID-19 related variables at assessment** | | | | | |
| **Sick leave** |  |  |  |  |  |
| 0% | 17 (15.3%) | 32 (19.5%) | 46 (19.5%) | 105 (40.5%) | 200 (26.0%) |
| 25% | 4 (3.6%) | 12 (7.3%) | 13 (5.5%) | 6 (2.3%) | 35 (4.5%) |
| 50% | 8 (7.2%) | 17 (10.4%) | 36 (15.3%) | 25 (9.7%) | 86 (11.1%) |
| 75% | 8 (7.2%) | 14 (8.5%) | 19 (8.1%) | 13 (5.1%) | 54 (7.0%) |
| 100% | 61 (55.0%) | 56 (34.1%) | 55 (23.3%) | 34 (13.1%) | 206 (26.8%) |
| Missing | 12 (10.8%) | 33 (20.1%) | 67 (28.4%) | 75 (29.0%) | 187 (24.3%) |
| **POTS** diagnosed post COVID 19 |  |  |  |  |  |
| Yes | 30 (27.0%) | 30 (18.3%) | 41 (17.4%) | 26 (10.0%) | 127 (16.5%) |
| Missing | 21 (18.9%) | 48 (29.3%) | 59 (25.0%) | 55 (21.2%) | 183 (23.8%) |
| **Symptoms,** at follow-up assessment |  |  |  |  |  |
| Mean (SD) | 12.4 (4.74) | 10.5 (5.06) | 9.03 (5.34) | 6.51 (4.57) | 8.98 (5.34) |
| ≤ 2 symptoms, n | 0 (0%) | 4 (2.4%) | 14 (5.9%) | 31 (12.0%) | 49 (6.3%) |
| ≥10 symptoms, n | 81 (73.0%) | 84 (51.2%) | 96 (40.7%) | 66 (25.5%) | 327 (42.5%) |
| **Outcome measures at assessment** | | | | | |
| **PCFS, pre COVID-19**/before illness onset |  |  |  |  |  |
| Median (IQR) | 0.0 (0.0 to 0.0) | 0.0 (0.0 to 0.0) | 0.0 (0.0 to 0.0) | 0.0 (0.0 to 0.0) | 0.0 (0.0 to 0.0) |
| Missing | 3 (2.8%) | 10 (6.5%) | 11 (4.9%) | 14 (5.7%) | 38 (4.9%) |
| **PCFS,** at assessment |  |  |  |  |  |
| Median (IQR) | 3.0 (3.0 to 3.0) | 2.0 (1.0 to 3.0) | 2 (1.3 to 3.0) | 1 (0.0 to 2.0) | 2.0 (1.0 to 3.0) |
| Missing | 2 (1.8%) | 5 (3.1%) | 2 (0.9%) | 5 (2.0%) | 14 (1.8%) |
| **EQ Visual Analogue Scale** |  |  |  |  |  |
| Mean (SD) | 34.8 (18.0) | 452 (20.0) | 52.0 (21.7) | 63.6 (20.2) | 51.8 (22.6) |
| Missing | 4 (3.6%) | 5 (3.0%) | 20 (8.5%) | 18 (6.9%) | 47 (6.1%) |
| **mMRC** dyspnoea score |  |  |  |  |  |
| Median (IQR) | 3.0 (2.0 to 4.0) | 2.0 (1.0 to 3.0) | 2.0 (1.0 to 3.0) | 1.0 (1.0 to 2.0) | 2.0 (1.0 to 3.0) |
| Missing | 6 (5.7%) | 5 (3.1%) | 15 (6.8%) | 16 (6.6%) | 42 (5.5%) |
| **Physical function**  Self-assessed | | | | | |
| **Frändin-Grimby, pre COVID-19,** before illness onset |  |  |  |  |  |
| Median (IQR) | 4.0 (4.0 to 5.0) | 5.0 (4.0 to 5.0) | 4.0 (4.0 to 5.0) | 4.0 (4.0 to 5.0) | 4.0 (4.0 to 5.0) |
| Missing | 2 (1.8%) | 2 (1.2%) | 6 (2.6%) | 3 (1.2%) | 13 (1.7%) |
| **Frändin-Grimby,** at assessment |  |  |  |  |  |
| Median (IQR) | 2.0 (2.0 to 3.0) | 3.0 (2.0 to 3.0) | 3.0 (2.0 to 3.0) | 3.0 (3.0 to 4.0) | 3.0 (2.0 to 3.0) |
| Missing | 2 (1.8%) | 1 (0.6%) | 6 (2.6%) | 3 (1.2%) | 12 (1.6%) |
| **Physical function**  Objectively Measured | | | | | |
| **SpO_2_ %,** at rest |  |  |  |  |  |
| Mean (SD) | 98.9 (1.4) | 98.8 (1.5) | 98.6 (1.6) | 98.7 (1.5) | 98.7 (1.5) |
| **Heartrate, beats/minute,** at rest |  |  |  |  |  |
| Mean (SD) | 82.2 (14.5) | 77.8 (13.0) | 80.0 (13.4) | 77.9 (13.3) | 79.2 (13.5) |
| **6MWT** test, % of predicted |  |  |  |  |  |
| Mean (SD) | 50.0 % (17) | 90.0 % (14) | 74.6 % (18) | 101.0 % (14) | 83.3 % (23) |
| **1MSTST** test, % of predicted |  |  |  |  |  |
| Mean (SD) | 36.2 % (14) | 68.0 % (19) | 50.5% (16) | 83.0 % (22) | 63.1% (25) |
| **Gripstrength**, % of predicted |  |  |  |  |  |
| Mean (SD) | 70.4 % (25) | 86.2 % (21) | 78.7% (23) | 97.5% (19) | 85.6% (24) |
| Missing, n (%) | 16 (14.4%) | 19 (11.6%) | 34 (14.4%) | 28 (10.8%) | 97 (12.6%) |
| **Pulmonary function** | | | | | |
| **Lungfunction** | | | | | |
| **FVC** % of predicted |  |  |  |  |  |
| Mean (SD) | 78% (16) | 83% (16) | 79% (17) | 85% (15) | 82% (16) |
| Missing | 11 (9.9%) | 10 (6.1%) | 24 (10.2%) | 21 (8.1%) | 66 (8.6%) |
| **FEV_1_** % of predicted |  |  |  |  |  |
| Mean (SD) | 78% (16) | 84% (17) | 80% (16) | 86% (15) | 82% (16) |
| Missing | 11 (9.9%) | 10 (6.1%) | 24 (10.2%) | 21 (8.1%) | 66 (8.6%) |
| **FEV1/FVC** ratio of predicted |  |  |  |  |  |
| Mean (SD) | 0.79 (0.07) | 0.79 (0.06) | 0.80 (0.08) | 0.78 (0.07) | 0.79 (0.07) |
| Missing | 11 (9.9%) | 13 (7.9%) | 24 (10.2%) | 22 (8.5%) | 70 (9.1%) |
| **MIP** % of predicted |  |  |  |  |  |
| Mean (SD) | 69% (0.3) | 96% (0.3) | 77% (0.3) | 107% (0.3) | 90% (0.3) |
| **Mental health, cognitive function and fatigue**  Self-assessed | | | | | |
| **PHQ-9** **depression symptom** (≥ 10) | 106 (95.5%) | 147 (89.6%) | 56 (23.7%) | 35 (13.5%) | 344 (44.7%) |
| **GAD-7 anxiety symptom** (≥ 10) | 68 (61.2%) | 100 (60.9%) | 3 (1.3%) | 5 (1.9%) | 176 (22.9%) |
| **MoCA** (<26) | 32 (28.8%) | 25 (15.2%) | 49 (20.8%) | 37 (14.3%) | 143 (18.6%) |
| **FSS fatigue severity** (≥ 4) | 74 (66.7%) | 94 (57.3%) | 114 (48.3%) | 95 (36.7%) | 377 (49.0%) |
| Missing | 35 (31.5%) | 60 (36.6%) | 93 (39.4%) | 106 (40.9%) | 294 (38.2%) |

Data are presented as numbers (%), mean (SD) or median (IQR). Some variables don’t have missing data since they are included in the clusteranalysis. Follow-up = number of days from illness onset to first assessment. Abbreviations: ICU=Intensive Care Unit. POTS=postural orthostatic tachycardia syndrome. PCFS= the post-COVID-19 Functional Status. mMRC= the modified Medical Research Council dyspnoea scale. SpO_2_=peripheral oxygen saturation. 6MWT=Six-Minute Walking Test. 1MSTST=1 minute sit-to-stand test. FVC=forced vital capacity. FEV_1_=forced expiratory volume in one second. MIP=Maximal Inspiratory Pressure. PHQ-9= the Patient Health Questionnaire-9. GAD-7=General Anxiety Disorder Questionnaire-7. MoCA= Montreal Cognitive Assessment test. FSS=Fatigue Severity Scale.
