# Supplementary material for: Health outcomes in hospitalised and non-hospitalised individuals after COVID-19, an observational, cross-sectional study
Source: Commun Med (Lond). 2025 Dec 4;5:512. doi: 10.1038/s43856-025-01251-5 (PMC12678783; doi:10.1038/s43856-025-01251-5)
Supplement: Supplementary file 8 — Supplementary Data 5 [file 43856_2025_1251_MOESM8_ESM.docx]

**Supplementary Data 5**

**All symptoms reported at follow up assessment, after COVID-19 and presented as prevalence by cluster 1 to 4 (n=770).**

|  | **Cluster 1**  **n=111** | **Cluster 2**  **n=164** | **Cluster 3**  **n=236** | **Cluster 4**  **n=259** |
| --- | --- | --- | --- | --- |
| **Symptoms,** prevalence at assessment n (%) |  |  |  |  |
| Dyspnoea | 87 (78) | 105 (64) | 153 (65) | 121 (47) |
| Fatigue | 80 (72) | 126 (77) | 152 (64) | 135 (53) |
| Joint pain | 64 (58) | 89 (54) | 123 (52) | 79 (31) |
| Cough | 55 (50) | 58 (35) | 94 (40) | 69 (27) |
| Paraesthesia | 63 (57) | 71 (43) | 92 (39) | 69 (27) |
| Chest pressure | 60 (54) | 73 (45) | 91 (39) | 62 (24) |
| Palpitations | 53 (48) | 55 (34) | 88 (37) | 72 (28) |
| Reduced fitness | 43 (39) | 65 (40) | 85 (36) | 72 (28) |
| Weight gain | 32 (29) | 44 (27) | 70 (30) | 58 (23) |
| Concentration | 49 (44) | 73 (45) | 68 (29) | 53 (21) |
| Headache | 40 (36) | 56 (34) | 68 (29) | 58 (23) |
| Dizziness | 45 (41) | 58 (35) | 63 (27) | 51 (20) |
| Memory issues | 53 (48) | 66 (40) | 61 (26) | 62 (24) |
| Intermittent fever | 26 (23) | 36 (22) | 60 (25) | 46 (18) |
| Muscular weakness | 36 (32) | 37 (23) | 55 (23) | 34 (13) |
| Insomnia | 43 (39) | 67 (41) | 53 (22) | 50 (19) |
| Weight loss | 34 (31) | 44 (27) | 52 (22) | 52 (20) |
| PEM (post exertional malaise) | 24 (22) | 35 (21) | 42 (18) | 31 (12) |
| Dysfunctional breathing | 23 (21) | 28 (17) | 41 (17) | 30 (12) |
| Chest pain | 14 (13) | 28 (17) | 37 (16) | 22 (9) |
| Brain fog | 28 (25) | 30 (18) | 37 (16) | 30 (12) |
| Other symptoms (not listed) | 14 (13) | 15 (9) | 32 (14) | 25 (10) |
| Nausea | 20 (18) | 15 (9) | 28 (12) | 25 (10) |
| Impaired vision | 14 (13) | 26 (16) | 28 (12) | 23 (9) |
| Ageusia | 25 (23) | 26 (16) | 26 (11) | 29 (11) |
| Anosmia | 26 (23) | 27 (16) | 26 (11) | 25 (10) |
| Blemishes | 17 (15) | 8 (5) | 25 (11) | 10 (4) |
| Urticaria | 19 (17) | 18 (11) | 23 (1) | 22 (9) |
| Walking difficulties | 13 (12) | 11 (7) | 22 (9) | 4 (2) |
| Appetite | 26 (23) | 31 (19) | 19 (8) | 20 (8) |
| Worried | 13 (12) | 29 (18) | 20 (8) | 13 (5) |
| Diarrhea | 16 (14) | 21 (13) | 20 (8) | 10 (4) |
| Tinnitus | 9 (8) | 15 (9) | 19 (8) | 15 (6) |
| Sensory sensitivity | 18 (16) | 17 (10) | 19 (8) | 13 (5) |
| Dysphagia | 10 (9) | 14 (9) | 18 (8) | 9 (4) |
| Voice impairments | 16 (14) | 16 (10) | 18 (8) | 10 (4) |
| Depressed | 12 (11) | 16 (10) | 18 (8) | 7 (3) |
| Reduced balance | 18 (16) | 14 (9) | 17 (7) | 17 (7) |
| Fainting | 10 (9) | 8 (5) | 16 (7) | 5 (2) |
| Tremor | 11 (10) | 9 (5) | 13 (6) | 11 (4) |
| Pain during deep breathing | 10 (9) | 9 (5) | 11 (5) | 15 (6) |
| Nightmares | 15 (14) | 12 (7) | 12 (5) | 7 (3) |
| Hair loss | 11 (1) | 11 (7) | 12 (5) | 13 (5) |
| Changed breathing pattern | 10 (9) | 13 (8) | 11 (5) | 9 (4) |
| Obstipation | 7 (6) | 8 (5) | 11 (5) | 6 (2) |
| Malaise | 6 (5) | 9 (5) | 11 (5) | 10 (4) |
| Anxiety | 11 (1) | 17 (1) | 10 (4) | 6 (2) |
| Urinary tract problems | 6 (5) | 4 (2) | 9 (4) | 15 (6) |
| Mood changes | 8 (7) | 11 (7) | 9 (4) | 9 (4) |
| Rigor | 3 (3) | 7 (4) | 10 (4) | 8 (3) |
| Abdominal pain | 8 (7) | 14 (9) | 6 (3) | 8 (3) |
| Swollen joints | 7 (6) | 5 (3) | 6 (3) | 9 (4) |
| Slow movements | 3 (3) | 3 (2) | 8 (3) | 2 (1) |
| Irregular menstruation | 5 (5) | 6 (4) | 5 (2) | 1 (0) |
| Hearing impairments | 3 (3) | 6 (4) | 4 (2) | 1 (0) |
| Susceptible to infections | 1 (1) | 4 (2) | 2 (1) | 2 (1) |
| Erectile dysfunction | 0 (0) | 1 (1) | 1 (0) | 0 (0) |
| Yawning | 1 (1) | 1 (1) | 0 (0) | 0 (0) |
| Seizures | 0 (0) | 1 (1) | 0 (0) | 0 (0) |
